# Myeloid PTP1B deficiency protects against atherosclerosis by improving cholesterol homeostasis through an AMPK-dependent mechanism

**DOI:** 10.1186/s12967-023-04598-2

**Published:** 2023-10-12

**Authors:** Helk Oliver, Dekeryte Ruta, Dawn Thompson, Sarah Kamli-Salino, Sam Philip, Heather M. Wilson, Nimesh Mody, Mirela Delibegovic

**Affiliations:** 1 Aberdeen Cardiovascular and Diabetes Centre, Institute of Medical Sciences, University of Aberdeen, Foresterhill, Aberdeen, AB25 2ZD UK; 2 Present Address: Department of Medicine III, Division of Nephrology and Dialysis, Medical University of Vienna, Währinger Gürtel 18-20, 1090 Vienna, Austria; 3 Grampian Diabetes Research Unit, JJR Macleod Centre, NHS Grampian, Foresterhill, Aberdeen, AB25 2ZD UK

**Keywords:** PTP1B, Atherosclerosis, Myeloid cells, Cholesterol metabolism

## Abstract

**Objective:**

Atherosclerosis is a chronic inflammatory process induced by the influx and entrapment of excess lipoproteins into the intima media of arteries. Previously, our lab demonstrated that systemic PTP1B inhibition protects against atherosclerosis in preclinical LDLR^−/−^ models. Similarly, it was shown that myeloid-specific PTP1B ablation decreases plaque formation and ameliorates dyslipidaemia in the ApoE^−/−^ model of atherosclerosis. We hypothesized that the relevant improvements in dyslipidaemia following modification of PTP1B activation may either result from changes in hepatic cholesterol biosynthesis and/or increased uptake and degradation by liver-resident macrophages. We examined this in animal models and patients with coronary artery disease.

**Methods:**

In this study, we determined the cholesterol-lowering effect of myeloid-PTP1B deletion in mice fed a high-fat high-cholesterol diet and examined effects on total cholesterol levels and lipoprotein profiles. We also determined the effects of PTP1B inhibition to oxLDL-C challenge on foam cell formation and cholesterol efflux in human monocytes/macrophages.

**Results:**

We present evidence that myeloid-PTP1B deficiency significantly increases the affinity of Kupffer cells for ApoB containing lipoproteins, in an IL10-dependent manner. We also demonstrate that PTP1B inhibitor, MSI-1436, treatment decreased foam cell formation in Thp1-derived macrophages and increased macrophage cholesterol efflux to HDL in an AMPK-dependent manner. We present evidence of three novel and distinct mechanisms regulated by PTP1B: an increase in cholesterol efflux from foam cells, decreased uptake of lipoproteins into intra-lesion macrophages in vitro and a decrease of circulating LDL-C and VLDL-C in vivo.

**Conclusions:**

Overall, these results suggest that myeloid-PTP1B inhibition has atheroprotective effects through improved cholesterol handling in atherosclerotic lesions, as well as increased reverse cholesterol transport.

*Trial registration* Research registry, researchregistry 3235. Registered 07 November 2017, https://www.researchregistry.com/browse-the-registry#home/registrationdetails/5a01d0fce7e1904e93e0aac5/.

**Supplementary Information:**

The online version contains supplementary material available at 10.1186/s12967-023-04598-2.

## Introduction

### PTP1B and atherosclerosis

Protein tyrosine phosphatase 1B (PTP1B) is an intracellular non-receptor tyrosine phosphatase with critical roles in glucose metabolism [1], energy expenditure [2], migration of immune cells [3] and the immune response [4]. Deletion of PTP1B globally in mice produced a lean phenotype that was hypersensitive to insulin and resistant to diet-induced and/or age-induced obesity, as well as against insulin resistance from chronic inflammation in white adipose tissue [5].

The actions of PTP1B are mediated through its involvement in an abundant list of signalling cascades, of which the most prominent examples are the insulin-, JAK/STAT and leptin pathways [6–8]. Increased PTP1B expression and/or activity have been linked to several pathological conditions including cancer, diabetes and, more recently, atherosclerosis [9–13].

We previously demonstrated that MSI-1436, a PTP1B allosteric inhibitor, protects against the development of atherosclerotic plaques in the LDLR^(−/−)^ model of atherosclerosis, on a high-fat diet. While this was associated with improved glucose maintenance, increased phosphorylation of aortic Akt/PKB and AMPKα, as well as an ameliorated systemic lipid profile, the precise mechanisms resulting in these treatment effects is not yet fully understood [9]. We also demonstrated that selective deletion of myeloid-PTP1B prevents severe atherosclerosis in the ApoE^(−/−)^ mouse model of atherosclerosis [14]. This was associated with the same beneficial alterations in aortic insulin signalling as seen in systemic pharmacological inhibition of PTP1B as well as increased circulating levels of anti-inflammatory IL10 through hyperphosphorylation of STAT3 [9, 15]. This genotype also demonstrated resistance to high-fat diet induced hypercholesterolemia and insulin resistance in the absence of body weight effects, suggesting a direct beneficial metabolic effect of myeloid-PTP1B ablation in the context of dyslipidaemia-induced atherosclerosis [14]. Whilst hepatic deletion of PTP1B results in downregulation of lipogenic genes, it is not immediately obvious from existing literature how alterations in PTP1B levels in myeloid cells can result in such striking effects on lipid- and glucose-metabolism, thus warranting further investigation [16].

### The role of myeloid cells in plaque formation

Macrophages are key myeloid cells that play a crucial role in atherosclerotic plaque formation due to their ability to infiltrate into the arterial wall in response to local cholesterol accumulation, where they take up oxidised lipoproteins and promote inflammation [17]. Macrophages are heterogeneous cells and can be broadly classified into a pro-inflammatory M1 macrophage and an inflammation-resolving M2 phenotype, although these phenotypes have been defined in vitro and represent extreme states and do not accurately reflect the heterogeneity of activated macrophage populations in vivo [18].

In the context of atherosclerosis, M1-like macrophages are activated by, for example, oxidised LDL, necrotic cells, inflammatory cytokines and hypoxia. They drive atherogenesis and plaque instability through their inflammatory role and release of matrix degrading enzymes [19]. By contrast, M2-like macrophages promote resolution of atherosclerosis and increase plaque stability through tissue remodelling and repair as well as their ability for cholesterol efflux and efferocytosis [20].

Excess uptake of lipids by macrophages and subsequent foam cell formation is an important driver of plaque progression. In principle, macrophages can counteract this through export of excess lipids to high density lipoproteins (HDL) in a process called reverse cholesterol transport (RCT). In hyperlipidaemic humans the rate of lipoprotein influx typically exceeds the clearing capacity of tissue macrophages. Consequently, a goal of drug development has been to discover a means of lowering serum lipid levels and restoring lipid homeostasis in intra-arterial macrophages in order to prevent foam cell formation and induce plaque resolution [17].

### Kupffer cells

Kupffer cells (KC) are resident liver macrophages and are involved in the pathogenesis of chronic liver inflammation under conditions of pathologically increased lipid deposition (NAFLD) [21]. Furthermore, Kupffer cells play an important role in the uptake and degradation of lipoproteins in the liver: under physiological conditions approximately one third of all hepatic cholesterol clearance, and up to 70% of all LDL clearance, takes place in KCs [22]. The affinity of human KCs for lipoproteins was shown to be 18-fold higher when compared to liver parenchymal cells, which enables KCs to participate in cholesterol degradation to a relevant extent despite the fact that they only contribute 1–5% to total liver cell mass [23, 24]. The molecular mechanisms regulating the lipid uptake in KCs remain largely unclear; however, a study performed in rats demonstrated that hypercholesterolaemia leads to an increase of scavenger receptor class B type 1 (SR-B1) expression in KCs, possibly in an LXR-dependent manner [25, 26]. Crucially, Kupffer cells are affected by the LysM-Cre promoter although they are not of direct myeloid origin but are descendants of erythro-myeloid progenitors of the yolk-sac wall [27, 28].

Based on these findings we hypothesized that myeloid-PTP1B inhibition may ameliorate systemic atherosclerosis by improving the balance of cholesterol influx- and clearance from early lesions and by enhancing the clearance of lipoproteins towards biliary excretion through KCs. To investigate this further, we performed a series of experiments in the LysMPTP1B ^(−/−)^ genotype as well as in cell lines and primary cells, focussing on changes in lipid- and lipoprotein metabolism.

## Methods

### Animal studies and ethics statement

All animal procedures were performed under a project license approved by the U.K. Home Office under the Animals (Scientific Procedures) Act 1986 (PPL 60/3951) and were approved by the University of Aberdeen Ethics Review Board under the UK Home Office project licenses P94B395E0. All staff involved in the mouse experiments hold UK Home Office personal licenses and had been trained to competent standards before any procedures were performed. Eight-week-old male and female mice expressing Cre under the LysM promoter (LysMPTP1B^(−/−)^) as well as wild type (WT) littermate mice were bred in-house and maintained at 22–24 °C on 12-h light/dark cycle with free access to food/water [29]. Following 2 weeks of acclimatization time, mice were placed on high-fat diet (HFD) (42% from fat, 0.2% cholesterol, Envigo, Huntingdon, U.K., as previously described) for 13 weeks and weighed weekly with ad libitum access to food [9]. Glucose tolerance tests were performed at week 13 and body composition was measured at weeks 7 and 13, as described below.

#### Terminal procedures

At the end of week 13 on HFD, mice were fasted overnight prior to culling by CO_2_ inhalation and subsequent cervical dislocation. Tissues for subsequent qPCR analysis were frozen in liquid nitrogen and stored at –80 °C until needed.

Trunk-derived blood was collected into a BD Microtainer SST Tube (BD Biosciences, CA, USA). To evaluate serum lipid levels in a fasted state, mice were fasted for 16 h prior to terminal procedures. Blood was allowed to coagulate at RT for 30 min and subsequently stored at 4 °C before centrifuging at 7500 rpm for 15 min at 4 °C. In order to allow for exact lipid measurements, serum samples remained unfrozen and were stored at 4 °C for experimental processing the following day.

#### Measurement of circulating serum lipids

Total serum cholesterol-, LDL-C/VLDL-C, HDL-C and triglyceride concentrations were measured by a commercially available assay in accordance with instructions provided by the manufacturer (MAK043, MAK045 and MAK266, Sigma Aldrich). Assay readings were measured using a FLUOstar^®^ Omega multi-mode plate reader using spectrophotometer (BMG Labtech, Ortenberg, Germany).

#### Glucose tolerance tests

Mice were fasted overnight prior to commencement of glucose tolerance tests (GTTs). Briefly, baseline glucose levels were sampled from tail blood using glucose meters (AlphaTRAK, Abbott Laboratories). Subsequently, mice were injected I.P. with 20% glucose (w/v) and blood glucose measured at 15, 30, 60 and 90 min post-injection.

#### Body fat mass composition

Body composition of each mouse was analysed using an Echo MRI-3-in-1 scanner (Echo Medical Systems).

#### Kupffer cell lipoprotein uptake assay

Murine Kupffer cells were isolated using a protocol modified from Bourgognon et al. [30]. Kupffer cells were isolated from three 12 week old WT- and LysMPTP1B ^(−/−)^ animals each and plated into 96-well black plates (ThermoFisher Scientific) at a density of 50 k cells per well in 200 µl of DMEM medium supplemented with 10%FBS. After allowing the cells to recover for 48 h, the cells were washed and serum-starved overnight. Cells were then stained with SYBR green (ThermoFisher scientific), diluted 1:1000 in serum-free DMEM media, for 15 min with a single well remaining unstained as a blank control. Subsequently, cells were washed 3 times in serum free media. Then, dilLDL-C and dilVLDL-C (ThermoFisher Scientific) were diluted in serum free media to a respective final concentration of 15 µg/ml. Cells were covered with lipoprotein buffers for 4 h with a single well with normal serum free media as a blank control. Finally, all media was removed, cells were washed 3 times in PBS + 0.3%BSA and fluorescence was read out at the appropriate wavelengths. Blank-corrected fluorescence intensity values for DiI-labeled lipoproteins were normalized to blank-corrected fluorescence intensity values for SYBR green to calculate relative lipoprotein uptake.

### Cell line experiments

Thp1 cells are a monocyte-like cell line derived from leukaemia (ATCC#TIB-202)) and are a widely used cell line in macrophage research in the context of cardiovascular disease as Thp1-derived macrophages closely resemble the pathophysiology of in atherosclerotic plaques [31, 32]. The cells were maintained at a density of 300–1000 k cells per ml as a cell suspension in in RPMI-1640 medium with 25 mM glucose (Gibco, Thermo Fisher Scientific) supplemented with 10% foetal bovine serum and at 37 °C, 5% CO_2_. Prior to experimentation, Thp-1 cells were differentiated into a macrophage-like phenotype through incubation with protein kinase C activator phorbol myristate acetate (PMA) at 100 nM for 72 h. The newly differentiated Thp1 derived macrophages (M) were washed and allowed to recover in fresh media for 48 h before experimentation.

For some experiments, Thp1-derived macrophages were further transformed into foam cells (FC) by incubating them with 50 µg of oxLDL-C for 24 h under serum-starved conditions. To produce oxLDL-C, we obtained EDTA-free oxidized LDL-C (LeeBio) and oxidized it through exposure with CuSu4, as previously described by Morgan and Laeke [33]. Oxidation was verified by agarose gel electrophoresis. oxLDL was used for experimentation within 24 h following oxidation. In order to minimize variance, all experiments were performed from a single batch of original LDL-C solution.

#### Cholesterol efflux assay

Cellular cholesterol efflux was measured using a commercial assay kit (Abcam) in accordance with instructions provided by the manufacturer. Varying concentrations of MSI-1436 were added with the cholesterol acceptor (Apo-B depleted HDL-C acquired from a healthy donor).

#### Foam cell formation assay

Thp1 cells were plated into 96-well black plates (ThermoFisher Scientific) at a density of 50 K cells per well in 200 µl of RPMI 1640 medium supplemented with 10% FBS and were differentiated into macrophages as described earlier. After leaving the cells to recover for 48 h they were serum-starved overnight and stained with SYBR green (ThermoFisher scientific), diluted 1:1000 in serum-free RPMI 1640 media for 15 min with a single well remaining unstained as a blank control. Cells were then washed 3 times with serum free media. Then, DiIoxLDL-C (ThermoFisher Scientific) was diluted in serum free media to a final concentration of 15 µg/ml and cells were covered with 100 µl of DiIoxLDL-C buffer for 4 h with a single well with normal serum-free media as a blank control. Finally, all media was removed, cells were washed 3 times in PBS + 0.3%BSA and fluorescence was read out at the appropriate wavelengths in PBS (excitation: 544 nm, emission: 590 nm). Blank-corrected fluorescence intensity values for DiIoxLDL-C were normalized to blank-corrected fluorescence intensity values for SYBR green to calculate relative oxLDL-C uptake.

### Primary cell experiments

#### Ethics statement and inclusion criteria

A total of 60 volunteers consisting of 30 healthy volunteers (HV) and 30 patients with atherosclerosis (A) were recruited into this study. Ethical approval for this study was granted by the East Midlands—Leicester Central Research Ethics Committee (REC Ref 17/EM/0454).

##### Inclusion criteria

General Inclusion Criteria: Ability and willingness to provide informed consent, age above 18 and below 80 years.

Group A: Patients were be recruited with a medical history of manifest coronary artery disease.

Group B: Healthy Volunteers.

##### Exclusion criteria

Patients presenting with infection or other acute illness, cancer or autoimmune disease or are currently prescribed metformin, aspirin, metformin or immunosuppressant medication were excluded from the study.

Suitable subjects were identified through the Scottish Primary Care network and approached by the study team in writing. Once informed consent was provided, a medical anamnesis was taken using a case report form and the subjects hip-to-waist ratio was measured. Then, a 45 ml blood sample was taken into 4 × 9 ml EDTA-tubes.

#### Cell culture and experimental setup

Peripheral blood mononuclear cells (PBMCs) were isolated from the sample using Lymphoprep gradient (Stem cell technologies). The remaining cellular blood components were discarded. Then, monocytes were isolated from the PBMC suspension through positive selection using CD14 + microbeads (Miltenyi Biotech). The CD14 + cells were allowed to differentiate into macrophages in cell culture treated 6-well plates (ThermoFisher Scientific) for 7 days in RMPI-1640 media (Gibco, Thermo Fisher Scientific) supplemented with 10% serum isolated from the respective subject with the media being replaced after 3 days. After this time, the media was changed one for time and the cells were treated with 100 µl of oxLDL-C buffer with or without 3 µM of MSI-1436 for 4 h. At the end of the experiment, protein was harvested and used for immunoblotting as described below.

### Immunoblotting

Cells were lysed in 100 µl of ice-cold radioimmunoprecipitation assay (RIPA) buffer (10 mM Tris/HCl pH 7.4, 150 mM NaCl, 5 mM EDTA pH 8.0, 1 mM NaF, 0.1% SDS, 1% Triton X-100, 1% sodium deoxycholate with freshly added 1 mM NaVO_4_ and protease inhibitors) and lysates normalized to 1 µg per 1 µl after having been filtered through a syringe. Proteins were separated on a 4–12% bis–tris gel by SDS/PAGE and transferred on to nitrocellulose membrane. Membranes were probed for the following targets: p-JAK2 (Tyr1007/1008), p-STAT3 (Tyr705), SREBP1, PTP1B, p-AMPKα (Thr^172^). ABCA1, LXRα and GAPDH. Total protein levels for JAK2, STAT3 and AMPK were determined after stripping and reprobing of the membranes.

### RNA extraction and qPCR

Frozen liver tissues were lysed in TRIzol reagent (Sigma, U.K.) and RNA isolated using phenol/chloroform extraction as previously described [14]. Gene expression of intracellular low-density lipoprotein receptor (LDLR), very low-density lipoprotein receptor (VLDLR), CD38 and Lectin-like oxidized LDL receptor-1 was measured.

### Statistical analysis

All data were analysed using R version 4.12 (R Foundation for Statistical Computing, Vienna, Austria) with the following library packages: ggplot2 (Hadley Wickham, 2016), ggpubr (Alboukadel Kassambara, 2020), lmboot(Megan Heyman, 2019) and tidyverse (Hadley Wickham et al., 2019).

Data were analysed by residual bootstrap with a simulation of 1.000.000 replacement samples. A result where the sample mean was outside of 95% CI of the bootstrap distribution was considered statistically significant (corresponding to a p < 0.05).

## Results

### Ablation of myeloid-PTP1B improves the serum lipoprotein profile without affecting glucose sensitivity or body fat content

Myeloid-PTP1B deletion was previously shown to decrease circulating cholesterol levels in the ApoE background [14]. The hyperlipidaemic profile of the ApoE model is mainly driven by cholesteryl-ester rich VLDL remnants and thus exhibits a vastly different metabolic pathophysiology compared to the typical lifestyle-induced dyslipidaemia seen in humans, which is mainly caused by elevated levels of ApoB-100 rich LDL-cholesterol [34]. Consequently, we investigated if the effects of myeloid-PTP1B deficiency on cholesterol metabolism are reproducible in an otherwise genetically unaltered mouse model, i.e. in HFD induced dyslipidaemia.

To this end, we maintained LysMPTP1B^(−/−)^ and WT mice on HFD for 13 weeks. During this time, weight gain, body composition and glucose tolerance were assessed.

**Fig. 1:**
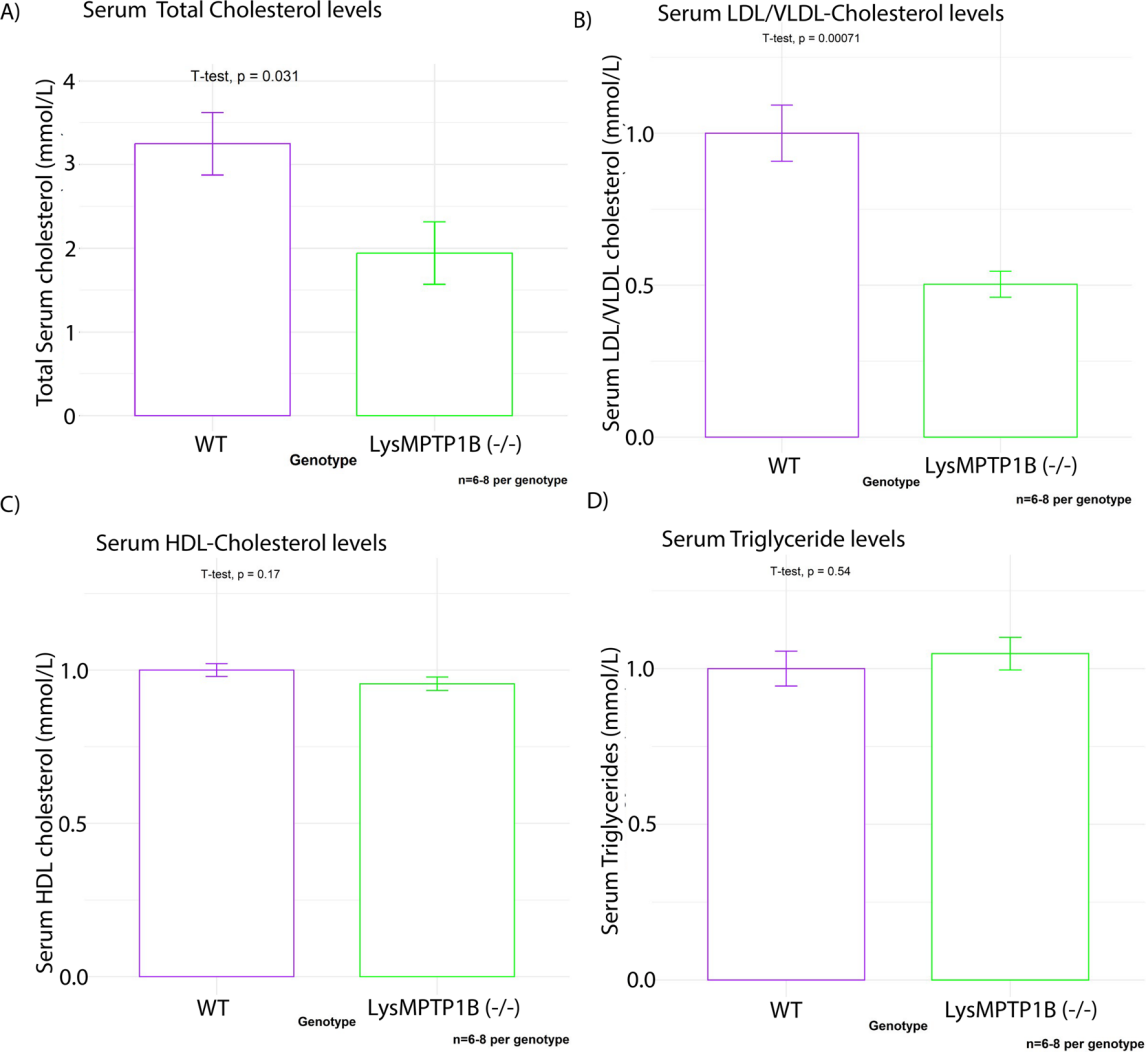
Myeloid PTP1B deletion results in significantly decreased circulating LDL/VLDL-C levels. Myeloid PTP1B ablation ameliorates high fat diet induced dyslipidaemia Blood was collected at terminal culls and serum was analyzed for circulating total cholesterol (**A**), LDL/VLDLcholesterol (**B**), HDL-cholesterol (**C**) and triglyceride levels (**D**). Data were normalized to the average of wild types for each respective sex and were then pooled due to the highly comparable relative differences between sexes and genotypes (Additional file 2: Fig. S2, Additional file 3: Fig. S3). Data are represented as mean ± S.E.M. and were analyzed by bootstrapped t-tests

Total circulating cholesterol levels of LysMPTP1B^(−/−)^ animals were consistently decreased in both sexes by approximately 50 percent compared to WT littermates (Fig. 1A). This was mainly driven by a specific decrease in the ApoB100-containing lipoprotein subfractions, i.e. LDL-C and VLDL-C (Fig. 1B), while there was no significant effect on HDL-C (Fig. 1C). Serum triglyceride levels (TG) (Fig. 1D) remained unaffected. Individual analysis of the lipid profile of male- and female mice separately is provided in Additional file 1: Fig. S1, Additional file 2: Fig. S2.

Physiological data on body weight and body composition is provided in Additional file 3: Fig. S3, Additional file 4: Fig. S4.

**Fig. 2:**
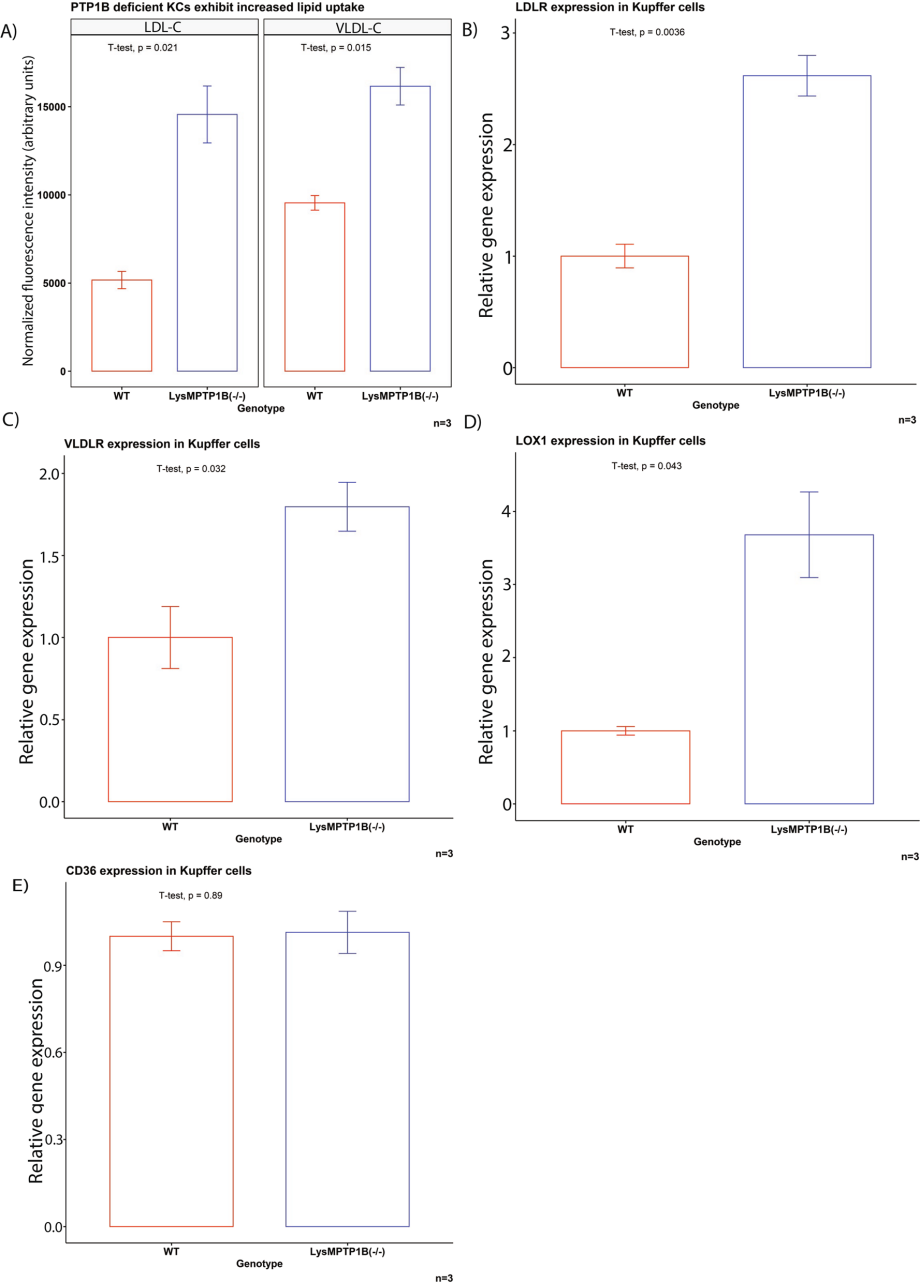
PTP1B deficient Kuppfer cells exhibit increased lipoprotein uptake through upregulation of lipoprotein receptors. **A** PTP1B deficient Kupffer cells exhibit increased uptake of LDL-C and VLDL-C. This correlates with increased gene expression levels of LDL-R, VLDL-R and LOX1 (**B**–**D**). CD36 expression remained unaffected (**E**). Data are represented as mean ± S.E.M. and were analyzed by bootstrapped t-tests

Systemic myeloid cells are not recognized as major direct regulators of systemic cholesterol homeostasis [35]. Consequently, we proceeded to investigate KCs, as they are liver tissue resident macrophages affected by the LysM promoter with known involvement in maintaining systemic lipoprotein homeostasis [21, 36, 37]. To this end, KCs were isolated from male LysMPTP1B^(−/−)^ and WT littermates and we assessed the uptake of DiI-labeled non-oxidised lipoproteins. PTP1B-deficient KCs exhibited a significantly increased uptake of LDL-C and VLDL-C (Fig. 2A), matching the alterations seen in the circulating lipid profile of LysMPTP1B^(−/−)^ animals, while HDL-C uptake remained unaffected. These findings correspond to a significantly increased expression of the specific lipoprotein receptors LDLR, VLDLR and LOX1 in KCs (Fig. 2B–D). However, the expression of the unspecific scavenger receptor CD36 remained unaffected (Fig. 2E).

Whilst we cannot exclude the existence of alterations in *de-novo* cholesterol synthesis as a consequence of myeloid-PTP1B ablation, qPCR analysis of major regulators of lipid synthesis did not reveal significant differences between genotypes (Additional file 5: Fig. S5).

### PTP1B inhibitor treatment increases specific cholesterol efflux and decreases foam cell formation

Foam cell formation within incipient atherosclerotic lesions is caused by an excessive accumulation of cholesterol in macrophages, exceeding the capacity of macrophages for lipid clearance through cholesterol efflux towards HDL. This represents the most direct way through which cells of myeloid origin can affect the progression and development of atherosclerosis [17].

We therefore hypothesized that treatment with a PTP1B inhibitor would have beneficial effects on cellular cholesterol homeostasis in macrophages. To evaluate this, we performed a cholesterol efflux assay as well as a foam cell formation assay in Thp1 cells treated with increasing concentrations of the specific PTP1B inhibitor, MSI-1436 (Trodusquemine). This specific compound was chosen due to its reported higher specificity compared to other available PTP1B inhibitors and had been utilized in previous in vivo experiments by our lab [9]. Furthermore, we assessed changes in protein phosphorylation levels in known targets of PTP1B to investigate foam-cell specific treatment effects [38].

**Fig. 3:**
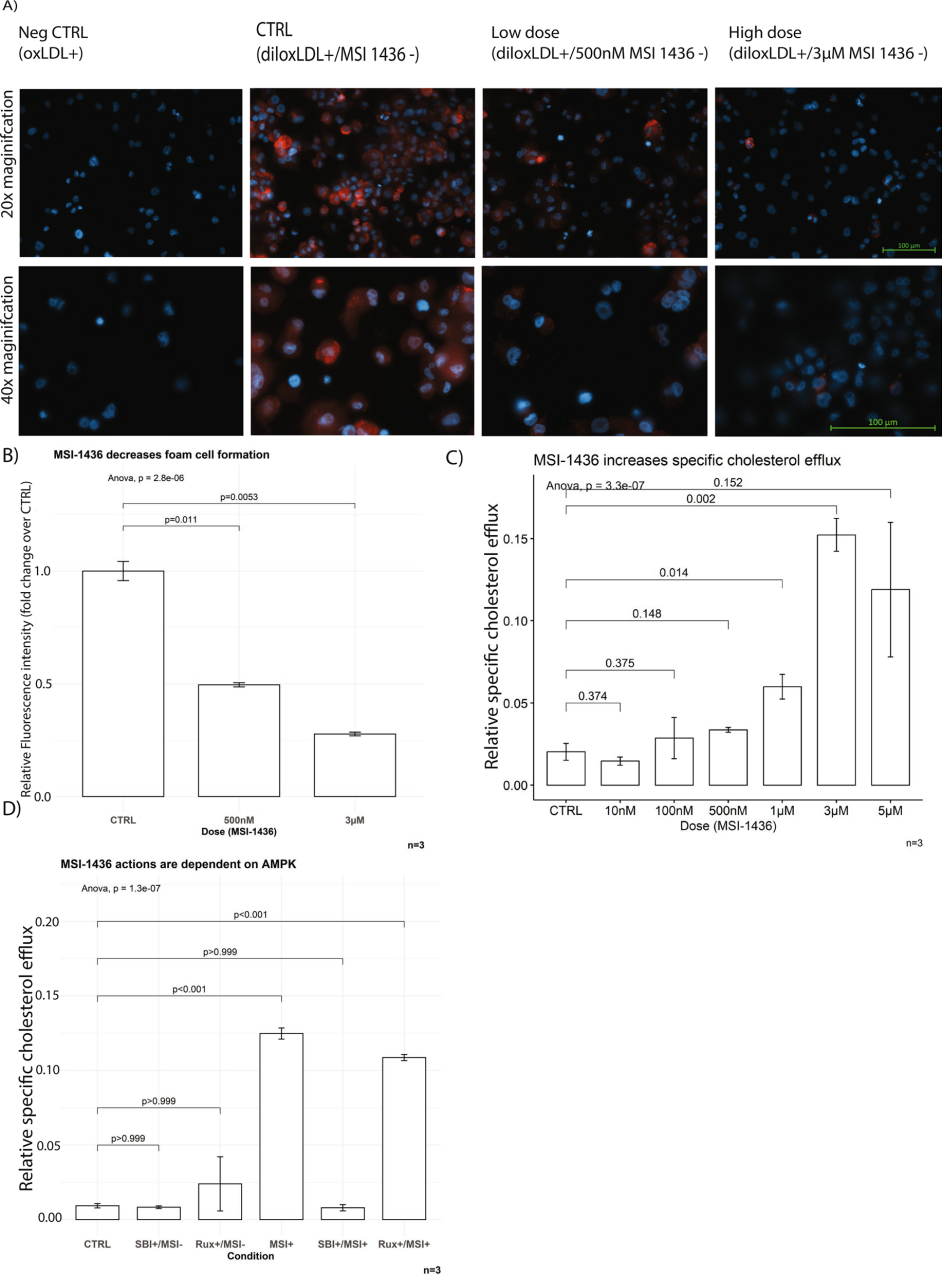
MSI-1436 inhibits foam cell formation and increases cholesterol efflux. MSI-1436 treatment decreases foam cell formation (**A**, **B**) and improves specific cholesterol efflux (**C**, **D**) in an AMPK- and dose dependent manner. Data are represented as mean ± S.E.M. and were analysed by bootstrapped ANOVA followed by Bonferonni-corrected multiple bootstrapped t tests in case of a significant omnibus test

In accordance with our primary hypothesis, MSI-1436 treatment significantly decreased the uptake of oxLDL by Thp1-derived macrophages (Fig. 3A, B) while also improving specific cholesterol efflux towards HDL from foam cells in a concentration-dependent manner (Fig. 3C). Treatment effects were dependent on AMPK activation, as the presence of the AMPK inhibitor SBI-0206965 (SBI) prevented effects on cholesterol efflux, whilst the presence of the JAK/STAT inhibitor Ruxolitinib (Rux) did not (Fig. 3D) [15, 39]. While the signalling targets of AMPK add plausibility to the conclusion that the effects of MSI-1436 in atherosclerosis are AMPK dependent, it needs to be pointed out that SBI-0206965 is not a specific inhibitor and has known off-target effects on other kinases [40].

**Fig. 4:**
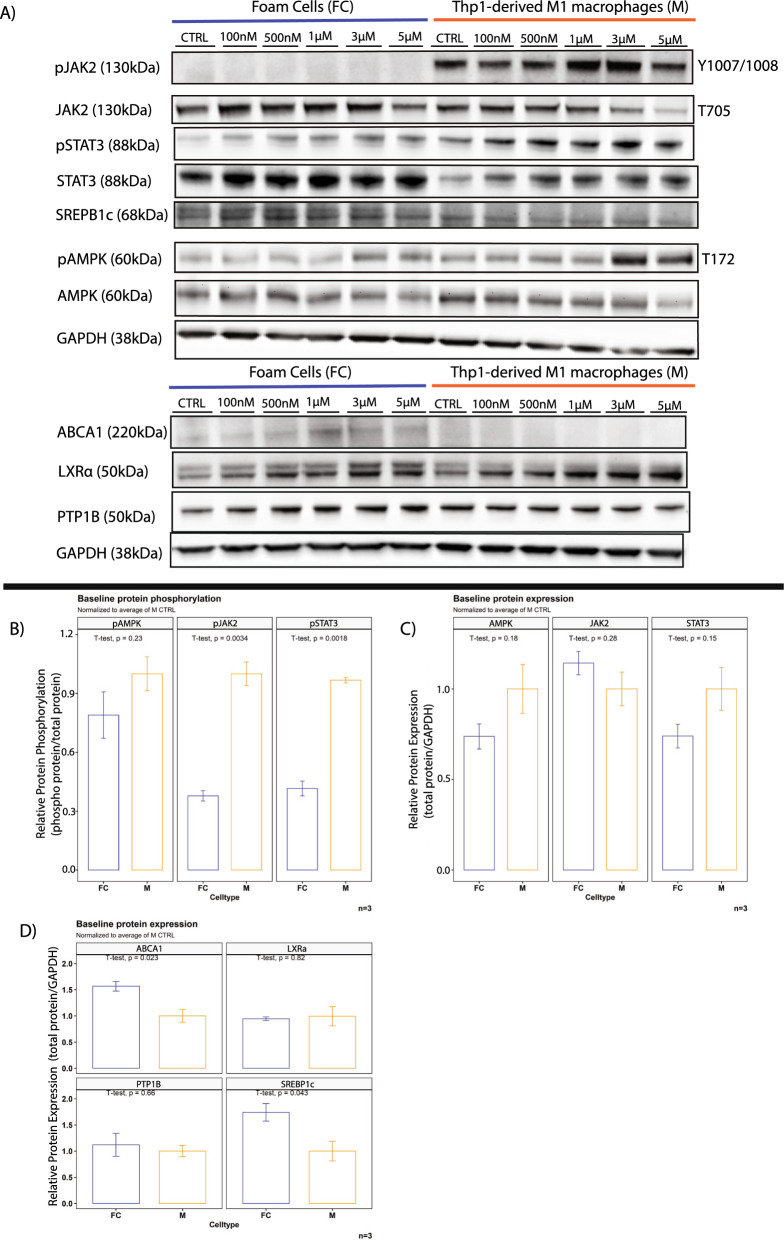
MSI-1436 ameliorates disturbances of AMPK signalling in foam cells. **A** Western blots from stimulation experiments with varying doses of MSI-1436 over a 3h time course B) Western blot quantification of whole protein levels and phosphorylation levels of the known PTP1B targets AMPK, JAK and STAT3 as well as total protein levels of ABCA1, SREBP1c, PTP1B and LXRa between foam cells and Thp1-derived macrophages. Quantification was performed using Image J software. **B** Quantification of baseline protein phosphorylation levels of AMPK, JAK2 and STAT3 between FC and M. **C** Quantification of baseline total protein levels of AMPK, JAK2 and STAT3 between FC and M. **D** Quantification of baseline total protein levels of ABCA1, LXRα, PTP1B and SREBP1c between FC and M. Data are represented as mean ± S.E.M. and were analyzed by bootstrapped t-tests

**Fig. 5:**
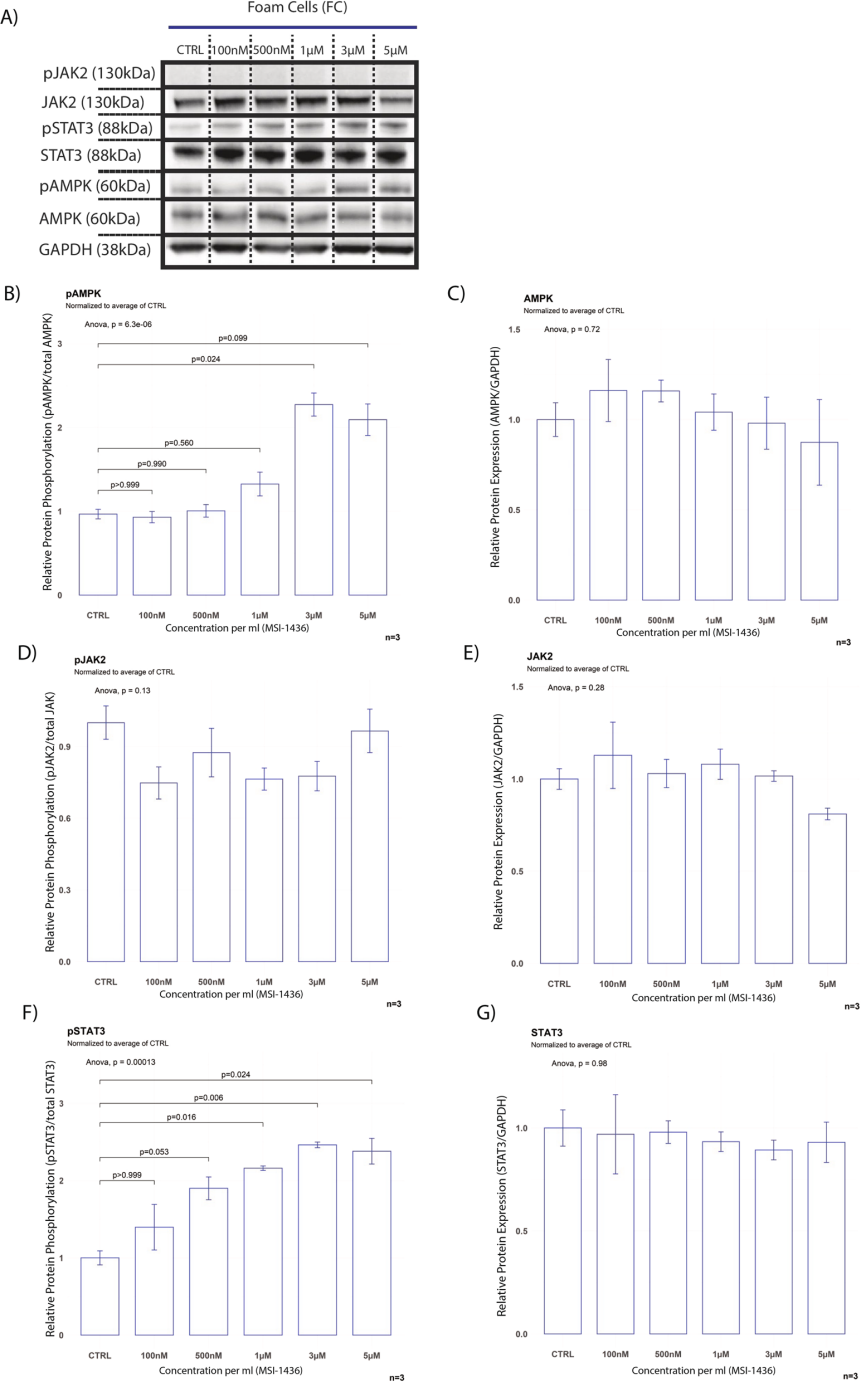
MSI-1436 leads to AMPK- and STAT3 hyperphosphorylation in foam cells with no effect on total protein levels. **A** Western blots from stimulation experiments in Thp-1 derived foam cells with varying concentrations of MSI-1436 over a 3h time course. **B**–**G** Western blot quantifications of pAMPK, AMPK, pSTAT3, STAT3, pJAK2 and JAK2 in Thp1-derived foam cells. Quantification was performed using Image J software. Data are represented as mean ± S.E.M. and were analyzed by bootstrapped ANOVA followed by Bonferonni-corrected multiple bootstrapped t tests in case of a significant omnibus test

**Fig. 6:**
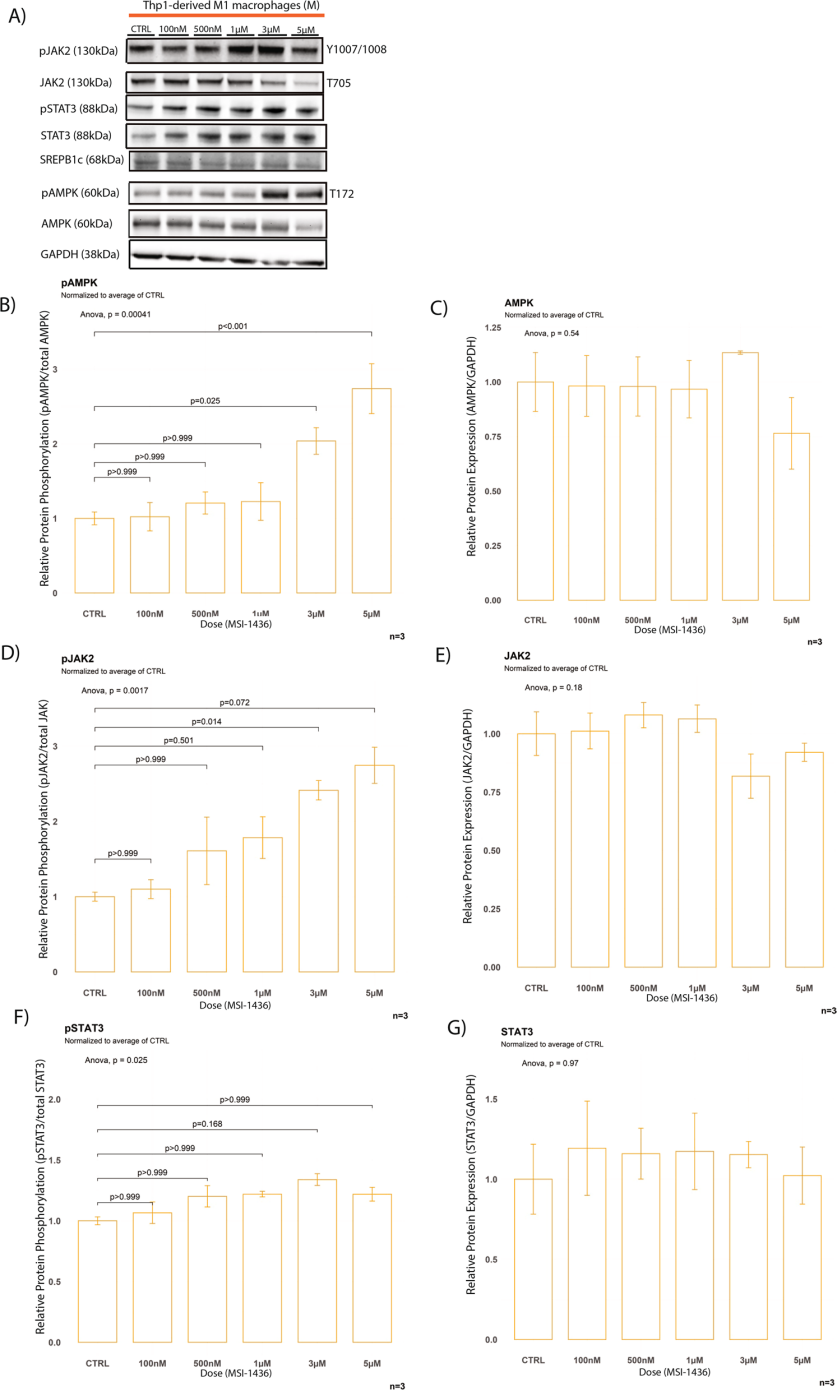
MSI-1436 induces hyperphosphorylation of AMPK and JAK2 in Thp1 derived macrophages. **A** Western blots from stimulation experiments in Thp1 derived macrophages with varying concentrations of MSI-1436 over a 3h time course. **B**–**G** Western blot quantifications of pAMPK, AMPK, pSTAT3, STAT3, pJAK2 and JAK2 in Thp1-derived non-lipid laden macrophages. Quantification was performed using Image J software. Data are represented as mean ± S.E.M. and were analyzed by bootstrapped ANOVA followed by Bonferonni-corrected multiple bootstrapped *t* tests in case of a significant omnibus test

STAT3 phosphorylation was previously identified as a major inducer of the inflammation-resolving M2 phenotype in macrophages by increasing IL-10 expression and was suggested as a potential mechanism for the protection of ApoE LysMPTP1B^(−/−)^ mice against atherosclerosis. While both STAT3 and JAK2 phosphorylation were decreased at baseline with no significant difference in pAMPK or total protein levels in FC compared to M, MSI-1436 rescued the STAT3 signal in FC without inducing hyperphosphorylation in M (Figs. 4A–C; 5F, 6F). MSI-1436 treatment resulted in hyperphosphorylation of JAK2 in M but failed to rescue JAK2 signalling in FC (Figs. 5D, E, 6D, E). FCs exhibited significantly increased levels of ABCA1 and SREBP1c at baseline compared to M as a result of their intracellular cholesterol burden (Fig. 4D) [41].

**Fig. 7:**
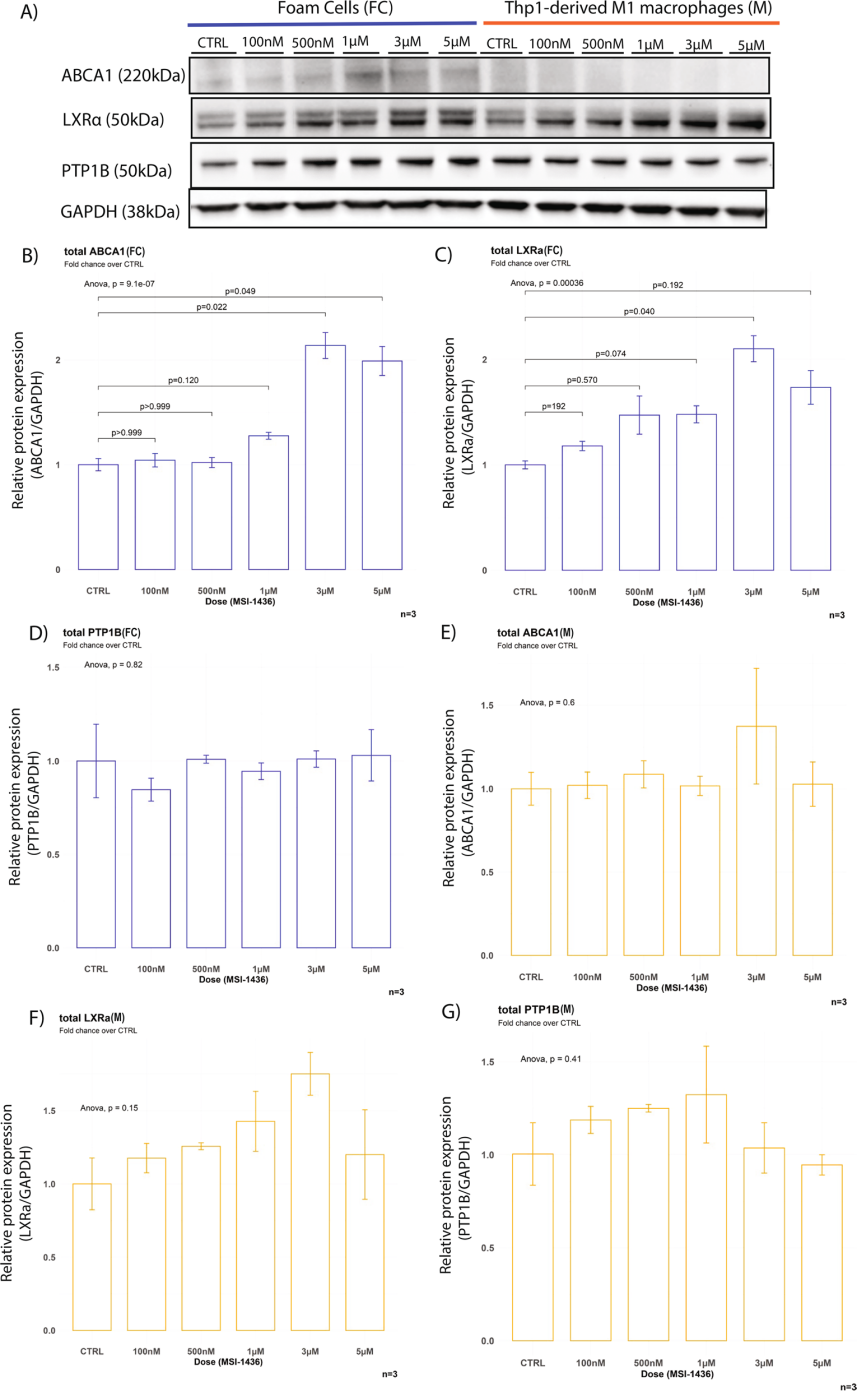
MSI-1436 treatment results in increased expression of LXRa/ABCA1 expression in foam cells. **A** Western blots from stimulation experiments in Thp1 derived macrophages with varying concentrations of MSI-1436 over a 3h time course. **B**–**D** Western blot quantifications of ABCA1, LXRa and PTP1B in Thp1-derived foam cells. **E**–**G** Western blot quantifications of ABCA1, LXRa and PTP1B in non-lipid laden Thp-1 derived macrophages. Quantification was performed using Image J software. Data are represented as mean ± S.E.M. and were analyzed by bootstrapped ANOVA followed by Bonferonni-corrected multiple bootstrapped t tests in case of a significant omnibus test

MSI-1436 treatment resulted in concentration-dependent hyperphosphorylation of AMPK with concomitantly increased protein levels of LXRα and ABCA1 in foam cells (Figs. 5A–C, 7A–C). While AMPK was also hyperphosphorylated in M as a consequence of MSI-1436 treatment, this did not result in increased LXRα or ABCA1 levels (Figs. 6A–C, 7A, E, F).

PTP1B levels did not change in response to MSI-1436 treatment in either F or M (Fig. 7D, G). Similarly, total AMPK, JAK2 and STAT3 remained unaltered by treatment in both cell types (Figs. 5C, E, G, 6C, E, G).

### MSI-1436 treatment induces signalling changes of key metabolic and inflammatory pathways in acute oxLDL-C challenge in macrophages

Based on the findings in Thp1-derived macrophages regarding beneficial effects of MSI-1436 on cholesterol handling in foam cells we aimed to investigate treatment consequences to key signalling pathways in acute oxLDL-C challenge in a study using primary human monocyte derived macrophages. This was done to assess the theoretical viability if pharmacological PTP1B inhibition could be effective in atherosclerosis prevention as well as reversal of existing plaques, as previously proposed by our lab [9].

#### Subject characteristics

A total of 30 healthy volunteers as well as 30 volunteers with known manifest coronary artery disease were recruited into the study. Subject characteristics are provided in Table 1.

**Table 1:** Clinical characteristics of the study population

Clinical characteristics (whole cohort)						
	Healthy volunteers			Patients affected with atherosclerosis		
	Male (n = 14)	Female (n = 16)	Overall (n = 30)	Male (n = 17)	Female (n = 13)	Overall (n = 30)
Mean age (years)	30.31 (± 7.21)	33.29 (± 8.46)	31.51 (± 7.90)	62.92 (± 11.08)	69.6 (± 9.41)	65.81 (± 8.01)
Mean height (cm)	183.57 (± 6.44)	167.83 (± 6.29)	175.16 ± (6.37)	174.83 (± 6.71)	163.29 (± 4.99)	169,82 (± 6,62)
Mean weight (kg)	84.78 (± 8.51)	66.14 (± 3.99)	74.84 (± 6.84)	80.71 (± 6.89)	62.19 (± 5.71)	72.68 (± 6.33)
BMI	23.22 (± 4.10)	23.19 (± 3.01)	23.21 (± 3.63)	26.03 (± 2.81)	23.82 (± 2.91)	25.07 (± 2.89)
Hip-to-waist ratio	0.91 (± 0.08)	0.83 (± 0.05)	0.87 (± 0.07)	0.99 (± 0.13)	0.84 (± 0.06)	0.93 (± 0.09)
Number of subjects with a history of smoking (n, %)	2	3	5	12 (71%)	10 (77%)	22 (73%)

Patients affected with atherosclerosis			
Type of CAD			
st.p. Major adverse cardiovascular event (n, %)	12 (71%)	6 (46%)	18 (60%)
Chronic coronary syndrome (n, %)	5 (29%)	7 (54%)	13 (43%)
*Mean years since first diagnosis*	5.71 (± 4.33)	3.47 (± 3.07)	4.73 (± 3.62)
*Number of subjects…*			
*Treated with antiplatelet therapy (n, %)*	13 (76%)	6 (46%)	19 (63%)
*Of which treated with dual antiplatelet therapy (n, %)*	4 (24%)	2 (15%)	6 (20%)
*With coronary stents (n, %)*	15 (88%)	9 (69%)	24 (80%)
*After coronary bypass surgery (n, %)*	6 (45%)	3 (23%)	9 (30%)
*With arterial hypertension (n, %)*	15 (88%)	10 (76%)	25 (83%)
*With diabetes mellitus (n, %)*	8 (47%)	4 (30%)	12 (40%)
*With metabolic syndrome (n, %)*	8 (47%)	3 (23%)	11 (37%)

Data are represented as mean (± SD) unless otherwise specified

#### MSI-1436 activates AMPK, JAK2 and STAT3 signalling in the presence of acute oxLDL-C challenge

In an initial step, we performed two-way ANOVAs as omnibus tests for main effects of treatment condition and group on changes in pAMPK, pSTAT3 and pJAK2. Since there was no statistically significant main effect for group and no significant interaction effect, the data from both groups were pooled and analysed collectively for effects of treatment condition in order to minimize the problem of multiple testing (Table 2). Data analysis of treatment effects for each group separately is available in Additional file 6: Fig. S6.

**Table 2:** 2-way ANOVA-statistics for main effects and interaction of treatment condition and group

Two-way ANOVA			
Target	Main effect: group	Main effect: treatment condition	Interaction
pAMPK	F(1,59) = 0.086, p = 0.7694	F(2,57) = 3.266, p = 0.0406	F(2,171) = 0.003, p = 0.9971
pJAK2	F(1,59) = 0.008, p = 0.8242	F(2,57) = 9.343, p = 0.0001	F(2,171) = 0.348, p = 0.7062
pSTAT3	F(1,59) = 0.209, p = 0.6483	F(2,57) = 4.150, p = 0.0174	F(2,171) = 0.119, p = 0.8876

**Fig. 8:**
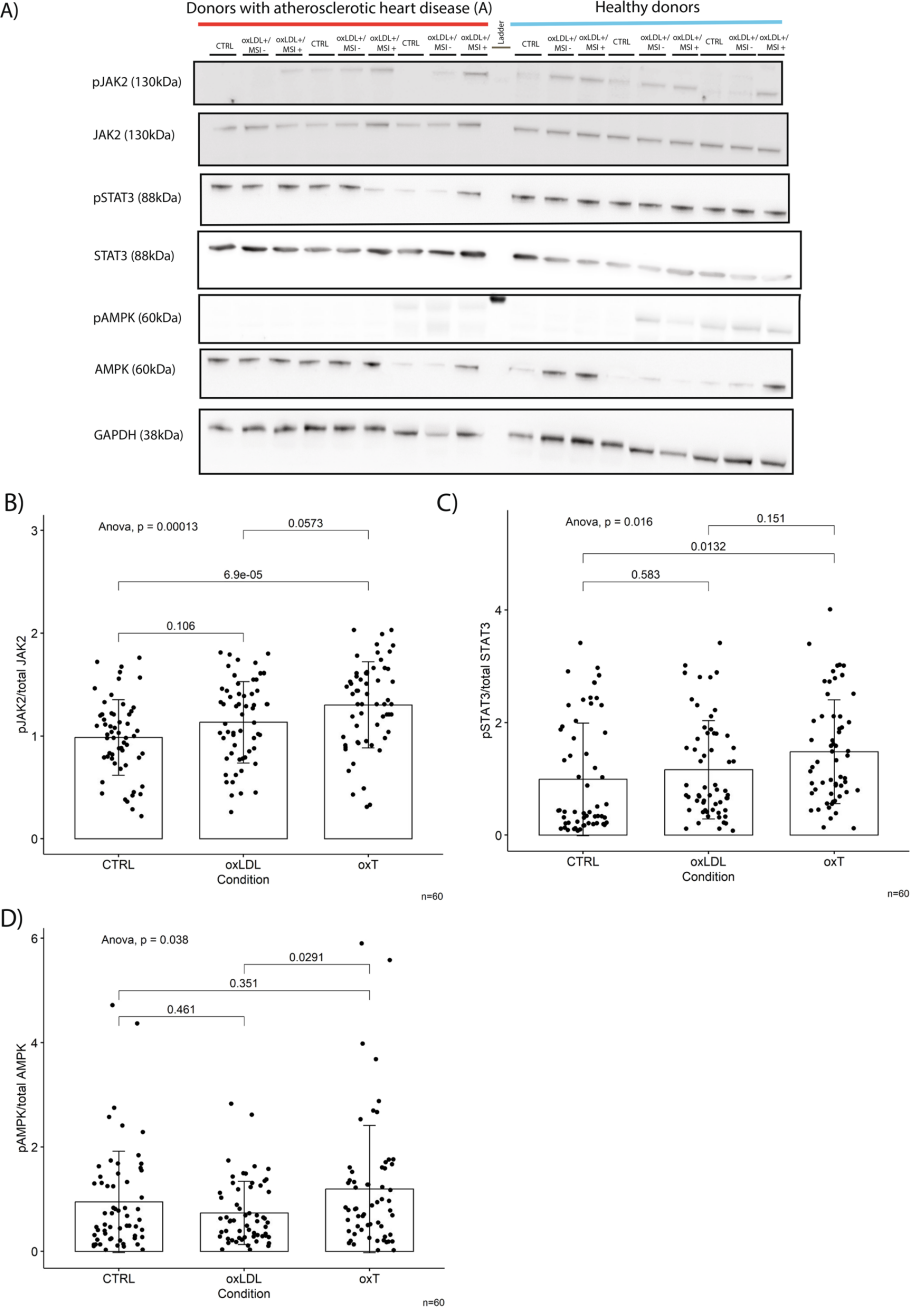
MSI-1436 induces hyperphosphorylation of AMPK, JAK2 and STAT 3 in primary human macrophages exposed to acute oxLDL-C challenge. **A** Western blots from stimulation experiments in primary human macrophages exposed to acute oxLDL-C stimulus in the presence or absence of MSI-1436 over a 3h time course. **B**-**D** Western blot quantifications of pAMPK, pSTAT3 and pJAK2. Quantification was performed using Image J software. Data are represented as mean ± S.E.M. and were analyzed by bootstrapped ANOVA followed by Bonferonni-corrected multiple bootstrapped t tests in case of a significant omnibus test

**Fig. 9:**
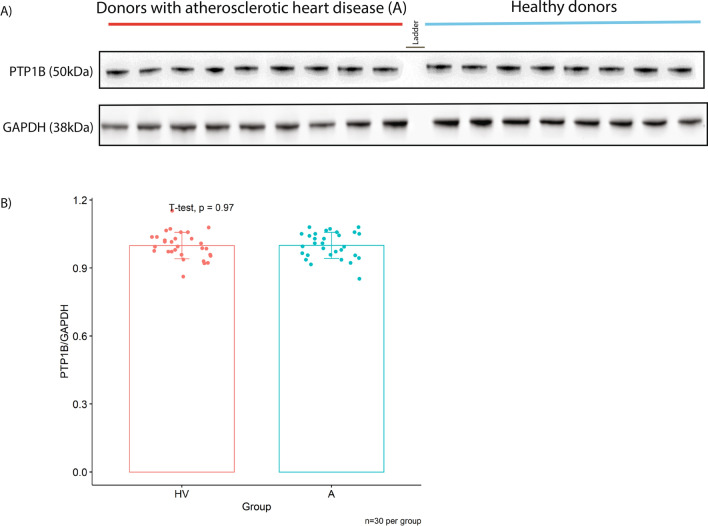
Macrophage PTP1B protein levels do not differ between patients with manifest heart disease and healthy volunteers. **A** Western blots unstimulated primary human macrophages from healthy volunteers (HV) and volunteers with manifest atherosclerotic heart disease (**A**) probed for PTP1B and GAPHD as a loading control. **B** Western blot quantifications of PTP1B normalized to GAPHD. Quantification was performed using Image J software. Data are represented as mean ± S.E.M. and were analyzed by bootstrapped t test

MSI-1436 treatment resulted in hyperphosphorylation of AMPK, JAK2 and STAT3 in the presence of oxLDL-C in a similar manner to what was seen in Thp1 derived macrophages without pro-inflammatory stimulus (Fig. 8). In agreement with our cell line data, we found no difference in macrophage PTP1B protein levels between groups (Fig. 9).

## Discussion

In this study we aimed to determine molecular mechanisms of the atheroprotective effects of PTP1B inhibition and myeloid-PTP1B deficiency [14]. Our results demonstrate the presence of three novel and distinct mechanisms: an increase in cholesterol efflux from foam cells, decreased uptake of lipoproteins into intra-lesion macrophages in vitro and a decrease of circulating LDL-C and VLDL-C in vivo.

We found that deficiency of myeloid PTP1B decreases circulating lipoproteins through increased uptake of LDL-C and VLDL-C into Kupffer cells, likely resulting in subsequent degradation and excretion through bile acid [23]. Mechanistically, this is probably a consequence of IL10 overexpression, which has been associated with protection against atherosclerosis in the LDLR^(−/−)^ model and is a hallmark characteristic of the LysMPTP1B^(−/−)^ genotype through a disruption in the negative IL10-STAT3 feedback-loop [15]. Indeed, a study by Chan et al. previously found that IL10 activates Kupffer cells to enhance uptake of circulating lipoproteins, resulting in a total decrease of up to 50% in human subjects [42]. PEGylated IL10 treatment specifically increased the uptake of LDL-C and VLDL-C into Kupffer cells but did not increase the affinity for HDL-C. Furthermore, ApoA1 levels remained unaltered [42]. Collectively, these findings suggest that the increased uptake of labelled lipoproteins into PTP1B deficient Kupffer cells reported here is driven by an IL10 dependent mechanism. It was previously demonstrated independently by various groups in different conditions and models that JAK2/STAT3 signalling and/or high levels of IL10 can induce an upregulation of lipoprotein receptors [43–45]. Another mechanism through which the STAT3/IL10 axis may influence macrophage lipid handling is by inducing PPARγ, which in turn was shown to increase the mitochondrial capacity for fatty acid oxidation [46, 47]. This in turn increases lipid turnover in cells and results increased presence of cell surface lipoprotein receptors [48]. Further investigation with a specific focus on Kupffer cell metabolism will be required to confirm the signalling mechanism underlying these changes and to validate increased biliary excretion of excess lipoproteins.

Our results do not demonstrate a dependence of the treatment effects of MSI-1436 on cholesterol efflux on JAK/STAT activation [15]. Indeed, JAK2 phosphorylation remained unaffected by MSI-1436 treatment even though JAK2 is an established target for PTP1B [7]. This may be explained by previous findings by Vaughan et al., which suggest that lipid-laden macrophages require the presence of a cholesterol acceptor, i.e., ABCA1/ApoA1 interaction, to induce JAK2 phosphorylation [49].

We were able to demonstrate that MSI-1436 increases cholesterol efflux through activation of the AMPK/LXR/ABCA1 axis. This effect was present in FC but not in M, which suggests that a substrate for cholesterol efflux (i.e. intracellular cholesterol) needs to be present for engagement of this pathway [50].

Effects of MSI-1436 on cholesterol efflux are not affected by the presence of Ruxolitinib, thus suggesting that the mechanism of action on cholesterol efflux is independent from JAK/STAT signalling (Fig. 3). PTP1B inhibition also resulted in a decreased uptake of lipoproteins by Thp1-derived macrophages. As both these changes improve cholesterol homeostasis in intra-lesion macrophages it can be proposed that these treatment effects are likely synergistic with each other [51].

Previous publications by our group reported on acute versus chronic treatment effects of pharmacological systemic PTP1B inhibition [9]. Based on these results questions remained if MSI-1436 can not only reverse atherosclerosis in a model of dyslipidaemia but also prevent plaque formation. Crucially, Bazzi et al. have recently demonstrated that oxLDL-C exposure results in a significant decrease of IL-10 production in macrophages, thus priming them towards the M1-phenotype and arresting them in a pro-inflammatory state. Our results seem to indicate that the presence of MSI-1436 can counteract these effects and ameliorate JAK2/STAT3 signalling, which may result in consecutive rescue of IL10 action [15].

Notably, STAT3 activity is commonly associated with detrimental effects in atherosclerosis [52, 53]. However, further results indicate a crucial role of AMPK activity in the regulation of STAT3 signalling in the context of atherosclerosis, thus making the interpretation of STAT3 activity in this context more complex [54–56]. Indeed, our results demonstrate hyperphosphorylation of both AMPK and JAK2/STAT3 signalling through MSI-1436 in acute oxLDL-challenge, which may result in a more favourable inflammatory phenotype and make beneficial effects of the compound in early atherosclerosis feasible. Indeed, in line with our findings a recent study by LeBlond et al. showed that acute oxLDL challenge induces AMPK activation in macrophages and induces autophagy pathways, which would be able to decrease foam cell formation. However, unregulated retention of chemically modified lipoproteins was shown to diminish these effects and the authors speculated that pharmacologic activation of AMPK could overcome this [57].

While we here provide evidence for the plausibility of this proposed effect, further research will be required to elucidate the role of signalling targets of PTP1B in early-stage atherosclerotic plaque formation. A possible further limitation of our results stems from the age difference between healthy volunteers and patients affected with atherosclerosis. However, we were unable to detect significant differences in signalling behaviour in acute response to oxLDL-C and MSI-1436 between groups. It appears unlikely that a theoretical effect from atherosclerotic disease would have been cancelled out by higher mean age [58].

PTP1B levels were not elevated in foam cells compared to non-lipid laden macrophages in Thp1 cell line experiments with similar results being found in primary cells from human patients with manifest coronary atherosclerosis. Thus, our results do not support a role of PTP1B overexpression in the pathogenesis of foam cell formation. This is in contrast to what was seen in a previous investigation of PTP1Bs role in insulin resistance in the adipose tissue of obese men, where PTP1B protein levels were increased [13]. Interestingly, a recent study by Clavier et al. reported that PTP1B expression in blood cells is significantly decreased in severe septic shock and predicts the development of multi-organ failure, thereby providing precedence for regulation of PTP1B during an extreme inflammatory response [59]. These findings are of particular interest, as previous mouse model experiments suggested that PTP1B inhibition- or deficiency were protective against mortality against bacterial sepsis. However, in the context of our own findings it is unclear if the decrease in PTP1B expression reported by Clavier et al. requires drastic immune responses—as can be found in septic shock—rather than chronic inflammation—as seen in atherosclerosis—to trigger these changes.

Notably, a recent study by Yang et al. reported that PTP1B expression was increased both in liver as well as primary macrophages in a mouse model of alcoholic liver injury, which appeared to be a direct toxic effect of alcohol that could be reproduced by in vitro stimulation. This increased PTP1B expression resulted in heightened levels of NFκB signalling, with consecutively increased secretion of pro-inflammatory cytokines and macrophage activation [60].

It seems likely that altered PTP1B expression is not a hallmark characteristic of general inflammation but rather a contextual response to specific stimuli. It is also important to highlight that whilst total levels of the protein are unaltered its activity may be upregulated. Indeed, MSI-1436 does not affect PTP1B expression levels but rather inhibits its activity, which would be in line with our findings of upregulated phosphorylation of PTP1B substrates, namely JAK2/STAT3/AMPK [61].

Increased AMPK activity has been convincingly associated with several distinctive beneficial effects on cardiovascular health in both, humans and rodents [62]. In agreement with our findings presented here, AMPK activation was shown to inhibit foam cell formation in vitro by regulating uptake of oxLDL through PP2A-induced inhibition of LOX-1 [63]. Our findings also complement the data presented in a previous publication by our lab where a single dose of MSI-1436 lead to robust hyperphosphorylation of the α1-subunit of AMPK and led to a decrease in aortic plaque area [9]. These findings are also in line with previous studies which demonstrated that PTP1B^(−/−)^ mice on high fat diet exhibit higher levels of AMPK activation than WT controls [64].

Our group previously reported that acute systemic MSI-436 treatment resulted in decreased presence of MCP-1 in aortic tissue [9]. Lowered levels of MCP-1 result in weaker attraction of monocytes into the atherosclerotic lesion [65]. This could represent a protective mechanism against atherosclerosis as this results in a lower number of intra-lesion macrophages available for foam cell formation. However, improved lipid clearance and thereby decreased presence of foam cells results in a less pro-inflammatory environment, which in turn decreases MCP-1 expression [66]. Consequently, lower levels of aortic MCP1 may be a surrogate of ameliorated inflammation rather than a mediator of a treatment effect of MSI-1436.

While we here focus on effects of decreased PTP1B activity in macrophage activity in the context of atherosclerosis, further research will be required to investigate the complex interplay of various types of immune cells in vascular calcification. In a process different from apoptosis and necrosis, neutrophils exposed to sterile stimuli such as oxLDL-C undergo lytic cell death and release their DNA as well as histones, which results in the formation of web-like structures called neutrophil extracellular traps (NETs). NETs contribute to the progression of atherosclerosis by activating immune cells, such as monocytes and dendritic cells, thereby enhancing the formation of foam cells. NETs can directly induce endothelial dysfunction and promote thrombosis, further worsening atherosclerotic complications [67].

When NETs activate macrophages and promote polarization into a pro-inflammatory phenotype, activated macrophages release cytokines and chemokines that further amplify neutrophil recruitment and activation, fostering a positive feedback loop [68, 69].

Inhibition or deficiency of PTP1B was shown to decrease the release of NETs in animal- and cell line experiments [70, 71]. However, PTP1B inhibition leads to increased phosphorylation of key signalling molecules, such as Akt and ERK, which were shown to promote NET formation [15, 72]. Control of NETosis may represent another mechanism by which PTP1B activity influences atherosclerosis progression, and this warrants further investigation.

### Conclusions

In conclusion, we have identified distinct and novel mechanisms through which myeloid-PTP1B inhibition may improve systemic cholesterol homeostasis, increase uptake of lipoproteins into KCs and contribute to plaque regression through decreased foam cell formation and increased reverse cholesterol transport. While other contributing treatment effects may exist and may remain yet undiscovered, the mechanisms proposed here may be sufficient to induce the plaque regression as they are likely synergistic with each other.

In summary, our findings highlight the potential attractiveness of myeloid PTP1B as a drug target [73]. Indeed, PTP1B inhibitors have previously entered phase I clinical trials in overweight and obese patients with metabolic syndrome. The research presented here highlights cells of the myeloid lineage and PTP1B as a potentially attractive target in dyslipidaemia and atherosclerosis.

### Supplementary Information

**Additional file 1: Figure S1.** Genotype-specific differences in serum lipid profiles (males only). Myeloid PTP1B ablation ameliorates high fat diet induced dyslipidaemia in male mice Blood was collected at terminal culls and serum analysed for circulating total cholesterol (**A**), LDL/VLDL-cholesterol (**B**), HDL-cholesterol (**C**) and triglyceride levels (**D**). Data are represented as mean ± S.E.M. and were analysed by bootstrapped t-tests.

**Additional file 2: Figure S2.** Genotype-specific differences in serum lipid profiles (females only). Myeloid PTP1B ablation ameliorates high fat diet induced dyslipidaemia in female mice Blood was collected at terminal culls and serum analysed for circulating total cholesterol (**A**), LDL/VLDL-cholesterol (**B**), HDL-cholesterol (**C**) and triglyceride levels (**D**). Data are represented as mean ± S.E.M. and were analysed by bootstrapped t-tests.

**Additional file 3: Figure S3.** Physiological data (male mice only). Myeloid PTP1B deletion does not affect adiposity in male mice. **A** Body weights were measured weekly with no significant differences in weight trends between genotypes. **B** GTTs revealed no significant differences in glucose tolerance between genotypes. **C** Body adiposity was evaluated using an Echo MRI 3-in-1 scanner where total body fat (**C**) and lean mass as well as the ratio (**C**, **D**) were determined (n = 3 per genotype). Data are represented as mean ± S.E.M. and were analysed by bootstrapped two-way ANOVA followed by Bonferonni-corrected multiple bootstrapped t tests in case of a significant omnibus test.

**Additional file 4: Figure S4.** Physiological data (female mice only). Myeloid PTP1B deletion does lead to detectable differences adiposity in female mice of our collective. **A** Body weights were measured weekly with no significant differences in weight trends between genotypes. **B** GTTs revealed no significant differences in glucose tolerance between genotypes. **C** Body adiposity was evaluated using an Echo MRI 3-in-1 scanner where total body fat (**C**) and lean mass as well as the ratio (**C**, **D**) were determined (n = 3 per genotype). Data are represented as mean ± S.E.M. and were analysed by bootstrapped two-way ANOVA followed by Bonferonni-corrected multiple bootstrapped t tests in case of a significant omnibus test.

**Additional file 5: Figure S5.** Effects of myeloid PTP1B deletion on gene expression of key hepatic regulators of lipid- and glucose metabolism. The LysMPTP1B ^(−/−)^ genotype exhibits moderately decreased gene expression levels of HMG-CoA-Reductase (**A**). There were no significant differences in expression levels of SREBP1, SREBP2 and FAS. **B**–**D** Data are represented as mean ± S.E.M. and were analysed by bootstrapped *t*-tests.

**Additional file 6: Figure S6.** Effects of MSI-1436 on human primary macrophages exposed to acute oxLDL-C challenge. Western blots exposed to acute oxLDL-C stimulus in the presence or absence of MSI-1436 over a 3h time course. **B**–**D** Western blot quantifications of pAMPK, pSTAT3 and pJAK2 from stimulation experiments in primary human macrophages. Data are provided for cells isolated from healthy volunteers (HV) and from volunteers affected with atherosclerotic heart disease (**A**) separately. Quantification was performed using Image J software. Data are represented as mean ± S.E.M. and were analyzed by bootstrapped ANOVA followed by uncorrected multiple bootstrapped *t*-tests (LSD) in case of a significant omnibus test.

## Data Availability

Primary data are available upon reasonable request.
