# Supplementary figures and images for: Myeloid PTP1B deficiency protects against atherosclerosis by improving cholesterol homeostasis through an AMPK-dependent mechanism
Source: J Transl Med. 2023 Oct 12;21:715. doi: 10.1186/s12967-023-04598-2 (PMC10568790; doi:10.1186/s12967-023-04598-2)

# Supplemental Figure 1

Genotype-specific differences in serum lipid profiles of male mice

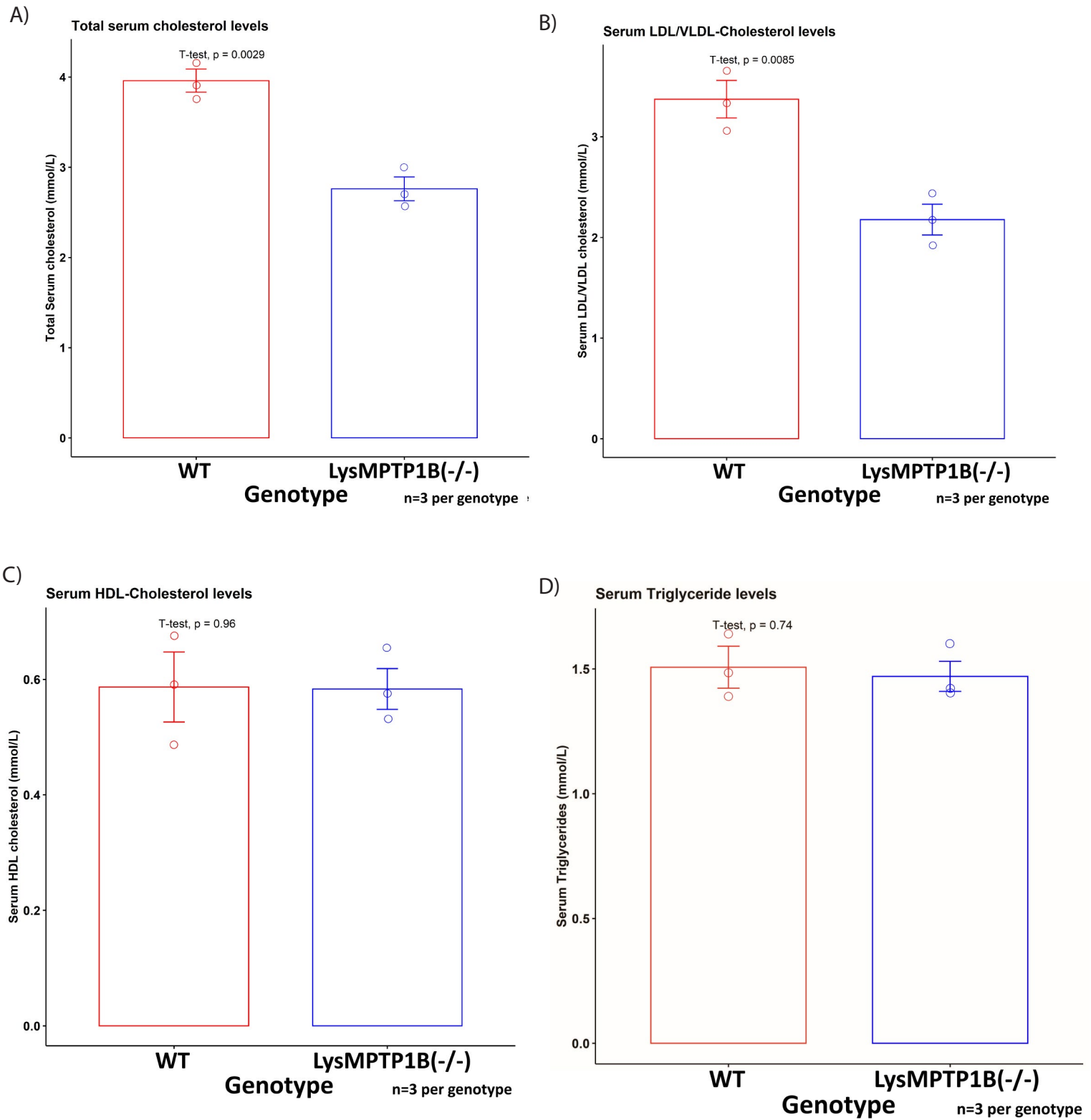

Supplement: Supplementary file 1 — Additional file 1: Figure S1. Genotype-specific differences in serum lipid profiles (males only). Myeloid PTP1B ablation ameliorates high fat diet induced dyslipidaemia in male mice Blood was collected at terminal culls and serum analysed for circulating total cholesterol (A), LDL/VLDL-cholesterol (B), HDL-cholesterol (C) and triglyceride levels (D). Data are represented as mean ± S.E.M. and were analysed by bootstrapped t-tests. [file 12967_2023_4598_MOESM1_ESM.pdf]

# Supplemental Figure 2

Genotype-specific differences in serum lipid profiles of female mice

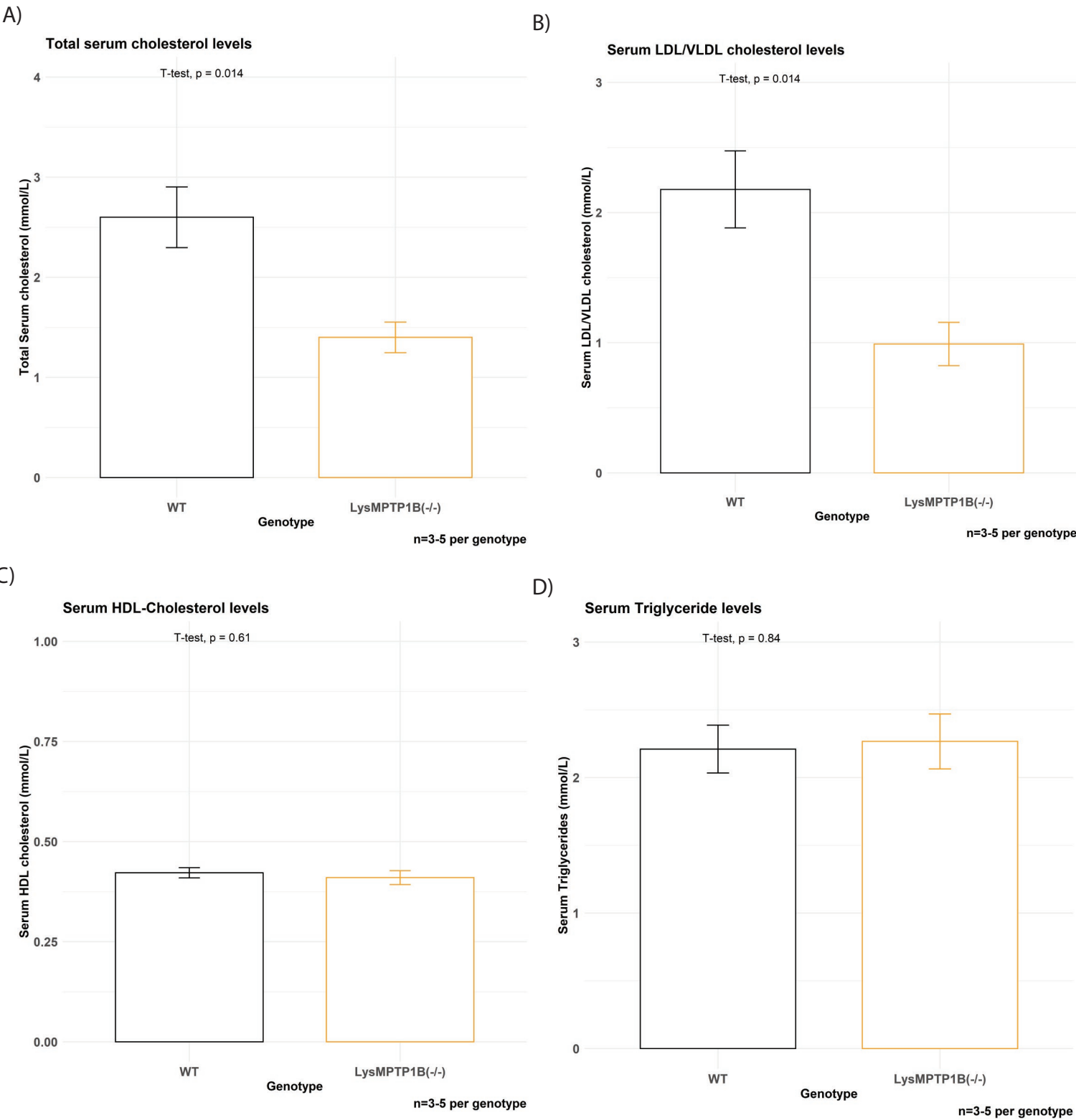

Supplement: Supplementary file 2 — Additional file 2: Figure S2. Genotype-specific differences in serum lipid profiles (females only). Myeloid PTP1B ablation ameliorates high fat diet induced dyslipidaemia in female mice Blood was collected at terminal culls and serum analysed for circulating total cholesterol (A), LDL/VLDL-cholesterol (B), HDL-cholesterol (C) and triglyceride levels (D). Data are represented as mean ± S.E.M. and were analysed by bootstrapped t-tests. [file 12967_2023_4598_MOESM2_ESM.pdf]
