# Supplementary material for: Myeloid PTP1B deficiency protects against atherosclerosis by improving cholesterol homeostasis through an AMPK-dependent mechanism
Source: J Transl Med. 2023 Oct 12;21:715. doi: 10.1186/s12967-023-04598-2 (PMC10568790; doi:10.1186/s12967-023-04598-2)

Supplemental Figure 4

Myeloid PTP1B deletion does not result in changes in adiposity or glucose tolerance in female mice

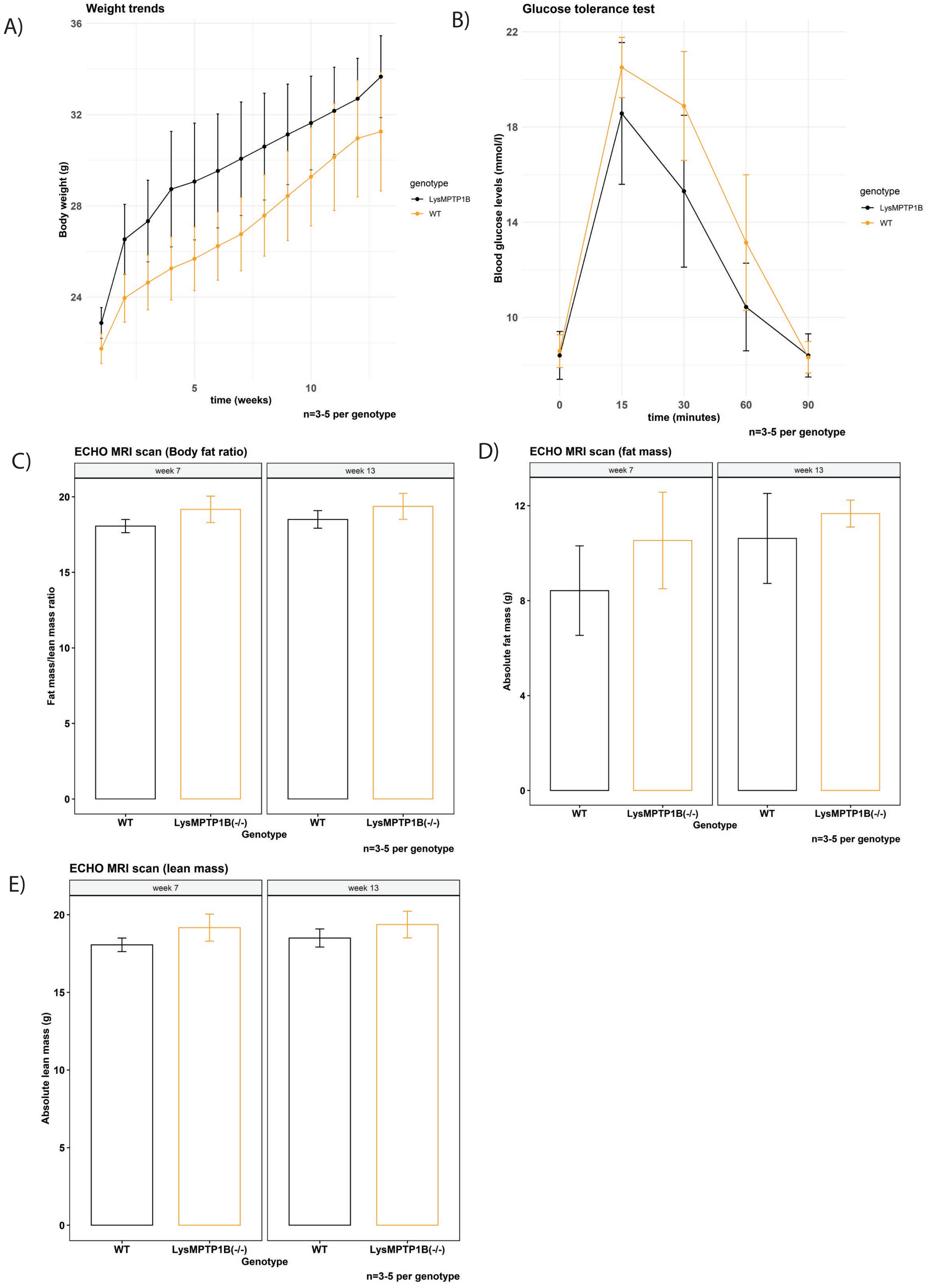

Supplement: Supplementary file 4 — Additional file 4: Figure S4. Physiological data (female mice only). Myeloid PTP1B deletion does lead to detectable differences adiposity in female mice of our collective. A Body weights were measured weekly with no significant differences in weight trends between genotypes. B GTTs revealed no significant differences in glucose tolerance between genotypes. C Body adiposity was evaluated using an Echo MRI 3-in-1 scanner where total body fat (C) and lean mass as well as the ratio (C, D) were determined (n = 3 per genotype). Data are represented as mean ± S.E.M. and were analysed by bootstrapped two-way ANOVA followed by Bonferonni-corrected multiple bootstrapped t tests in case of a significant omnibus test. [file 12967_2023_4598_MOESM4_ESM.pdf]
