# Supplementary material for: Myeloid PTP1B deficiency protects against atherosclerosis by improving cholesterol homeostasis through an AMPK-dependent mechanism
Source: J Transl Med. 2023 Oct 12;21:715. doi: 10.1186/s12967-023-04598-2 (PMC10568790; doi:10.1186/s12967-023-04598-2)

Supplemental Figure 5

Effects of myeloid PTP1B deletion on key regulators of hepatic lipid- and glucose metabolism

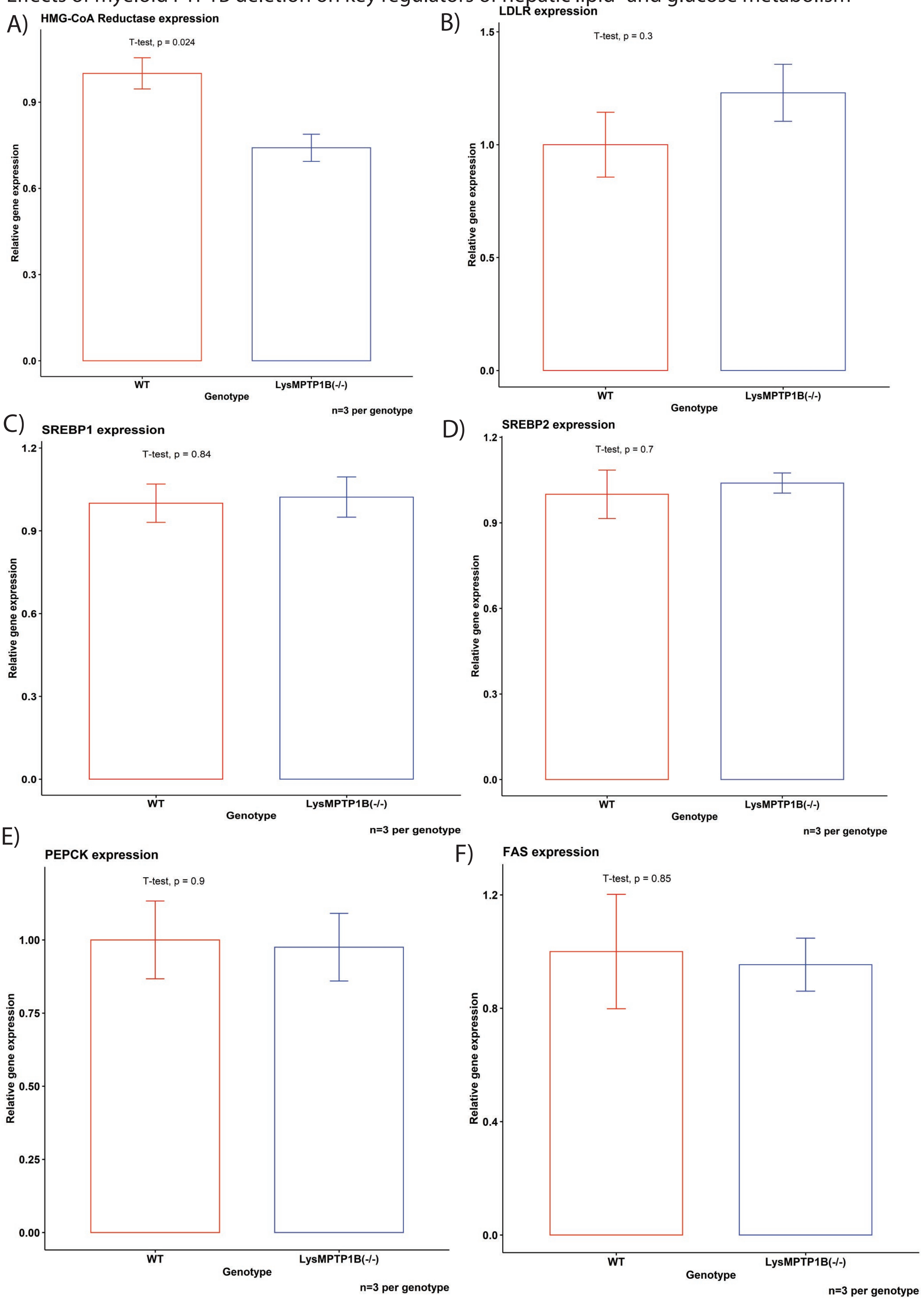

Supplement: Supplementary file 5 — Additional file 5: Figure S5. Effects of myeloid PTP1B deletion on gene expression of key hepatic regulators of lipid- and glucose metabolism. The LysMPTP1B (−/−) genotype exhibits moderately decreased gene expression levels of HMG-CoA-Reductase (A). There were no significant differences in expression levels of SREBP1, SREBP2 and FAS. B–D Data are represented as mean ± S.E.M. and were analysed by bootstrapped t-tests. [file 12967_2023_4598_MOESM5_ESM.pdf]
