# Supplementary material for: Myeloid PTP1B deficiency protects against atherosclerosis by improving cholesterol homeostasis through an AMPK-dependent mechanism
Source: J Transl Med. 2023 Oct 12;21:715. doi: 10.1186/s12967-023-04598-2 (PMC10568790; doi:10.1186/s12967-023-04598-2)

Supplemental Figure 6

MSI-1436 induces signalling changes in human monocytes comparable to those seen in the THP1 cell line

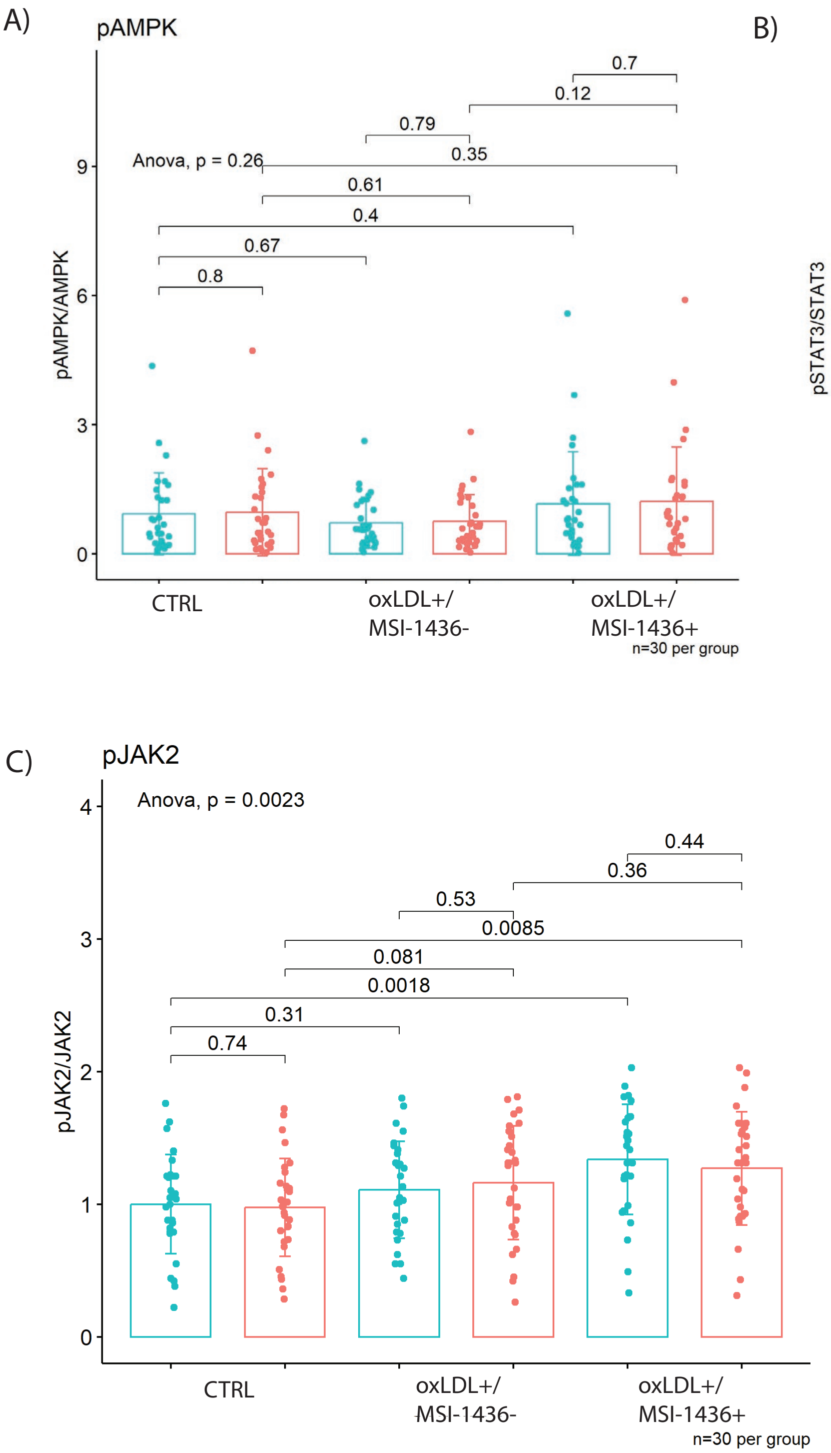

Supplement: Supplementary file 6 — Additional file 6: Figure S6. Effects of MSI-1436 on human primary macrophages exposed to acute oxLDL-C challenge. Western blots exposed to acute oxLDL-C stimulus in the presence or absence of MSI-1436 over a 3h time course. B–D Western blot quantifications of pAMPK, pSTAT3 and pJAK2 from stimulation experiments in primary human macrophages. Data are provided for cells isolated from healthy volunteers (HV) and from volunteers affected with atherosclerotic heart disease (A) separately. Quantification was performed using Image J software. Data are represented as mean ± S.E.M. and were analyzed by bootstrapped ANOVA followed by uncorrected multiple bootstrapped t-tests (LSD) in case of a significant omnibus test. [file 12967_2023_4598_MOESM6_ESM.pdf]
